# Medical Jurisprudence, Mania a Potu

**Published:** 1830

**Authors:** 


26. Medical Jurisprudence. *Mania a potu. Birdsell's case.* We should not again venture to refer to this monomaniac, had not the history of his trial attracted the attention of several of our brother Editors to a degree that evinces, that they have read it with interest. Our readers will recollect that in our July number, (1829) we ventured to express the opinion that *mania a potu*, like every other variety of mental alienation, should be received in our Courts of Justice, as an excuse for crime. We are happy to be able to say, that the learned Editors of the North American Medical and Surgical Journal, and of the Boston Medical and Surgical Journal, in quoting largely from our paper, have expressed their unqualified approbation of its sentiments. The latter have even entered into an extended and ingenious argument in support of the proposition.

The same history has attracted the attention of our associate profession, and we find in the 5th No. of the American Jurist and Law Magazine, published in Boston, an extended notice of this case, introduced by the following remarks:

An account of a trial before the Supreme Court of the state of Ohio, has just come into our hands, which, though different in some of its features, bears a strong resemblance to the case of Drew. In this case the point of law did not come directly before the court for a final decision. Whether the accused was at the time he committed the homicide, capable of discriminating between right and wrong, was a question, from all the circumstancea of the case to be left to thejury, and they having returned a verdict of *guilty*, the court upon a motion for a new trial did not think proper to interfere and disturb the verdict. The opinion of the court, however, so far as it can be gathered from incidental remarks made at the argument of counsel for a new trial, would seem to be at variance with the decision of the Circuit Court in the case of Drew. They say 'that they were not called upon to give an opinion, whether *Mania a potu* would, under any circumstances, be an excuse for the commission of a crime, but they feel no unwillingness to express their opinion, that if the insanity was the offspring of intemperance and the prisoner *knew* that intoxication would produce it, he could not plead it as an apology.' This intimation from the court, and the result of the trial, seem to have produced considerable excitement in that quarter of the country, and have drawn to the subject the attention of Dr. Drake, a distinguished medical professor, who, in the 'Western Journal of the Medical and Physical Sciences,' has stated the case and accompanied it with an elaborate discussion of the question 'whether *Mania a potu*, either temporary or protracted, be an excuse for crime.' He comes to the conclusion, the *correctness of which we cannot doubt*, 'that insanity of this kind ought in law to be an immunity from punishment.'

The case referred to in this extract is given in the Jurist, and being of the very highest authority, as occurring in one of the Circuit Courts of the United States, and under the adjudication of Mr. Justice Story, we shall transfer it entire to our columns. We doubt not it will be read with deep interest.

At the May Term, A. D. 1828, of the Circuit Court of the United States, Alexander Drew, commander of the whaling ship John Jay, was indicted and tried for the murder of his second mate, Charles F. Clark, while upon the high seas. It appeared in evidence that previously to the voyage, during which the fatal act took place, Drew had sustained a fair character, and was much respected in the town of Nantucket, where he belonged. It was proved that he was a man of humane and benevolent disposition, but that for several months he had been addicted to the use of ardent spirits, and for weeks during the voyage had drunk to excess; that he made a resolution to reform, and suddenly abstaining from drinking, he was seized with the delirium tremens, and that while under the influence of the disease he made an attack upon Clark, and gave him the stab of which he afterwards died.

The first witness who testified in the case was George Galloway, the cooper on board the ship. He stated that he joined the ship in the Pacific Ocean; that he found Capt. Drew to be an amiable man, kind to his crew and attentive to his business, but that he often indulged to excess in spirituous liquors. During the latter part of August, 1827, he had been in the habit of drinking very freely; that they spoke a ship from which Capt. Drew obtained a keg of liquor, and after he returned to his own vessel he drank until he became stupified; that soon after he recovered a little from his intoxication, and ordered the keg with its contents to be thrown overboard, and it was accordingly done. There being now no more liquor on board of the ship, and none to be procured, Capt. Drew, in two or three days, discovered signs of derangement. He could not sleep, had no appetite, thought the crew had conspired to kill him, expressed great fears of an Indian who belonged to the ship, called him by name when he was not present, begged he would not kill him, saying to himself he would not drink any more rum. Sometimes he would sing obscene songs and sometimes hymns, would be found alternately praying and swearing. In the night of the 31st of August, Drew came on deck and attempted to jump overboard, and when the witness caught hold of him he sunk down trembling, and appeared to be very weak. His appearance the next morning the witness described to be that of a foolish person. At seven o'clock in the morning of the first of September, the witness, Capt. Drew, Clark, and others, were at breakfast in the cabin, when Drew suddenly left the table and appeared to conceal something under his jacket which was on the transom in another part of the cabin. He immediately turned round to Mr. Clark and requested him to go upon deck; the reply of Clark was, 'When I have done my breakfast, sir.' Drew said 'Go upon deck, or I will help you,' and immediately took from the transom a knife which had been covered over by his jacket, and before another word was spoken by either, he stabbed Clark in the right side of his breast. Clark was rising from his chair at the time the knife struck him, and immediately fell upon the floor. He afterwards rose up and went upon deck alone. As the witness left the cabin, Drew cocked his pistol, pointed it at him, and snapped it, but it missed fire. Capt. Drew followed them upon deck, and addressing the chief mate, said 'Mr. Coffin, in twenty-four hours from this, the ship shall go ashore.' He was then seized, bound hand and foot, and a guard was stationed over him. His whole demeanor, for some time after, was that of an insane person. He would frequently call upon persons who were not on board, and who never had connexion with the ship. Some weeks after, when Drew first appeared to be in his right mind, he was informed of the death of Clark and its cause, he replied that he knew nothing about it, that when he awoke he found himself handsuffed, and that it all appeared to him like a dream. There had not been for months any quarrel or high words between Clark and Capt. Drew.

The second witness was Moses Coffin, the first mate of the ship. Coffin stated that Capt. Drew had been in the habit of drinking, and that it was by the order of Drew that the keg of spirits was thrown overboard. He recounted numerous instances in addition to those before stated, of frovolous complaints made by Drew, of his countermanding his orders, of his fear of being left alone, and his conversation with imaginary beings by whom he supposed himself surrounded, all going to prove physical weakness and alienation of mind. Though familiar with his habits, the witness had not, before this affair, snpposed him insane. With regard to Clark, the witness dressed his wound and took care of him. Two physicians at a Spanish port, which they reached soon after, gave it as their opinion that it was not dangerous, and that it would be well in a few days; but Clark himself had said, in describing his complaint to witness, that the wound caused an internal flow of blood. It healed externally before Clark expired.

At this stage of the proceeding, the Court asked the District Attorney if he expected to change the posture of the case. He admitted that unless upon the facts stated, the court were of opinion that this insanity, brought on by the antecedent drunkenness, constituted no defence for the act, he could not expect success in the prosecution. After some consultations, the opinion of the court was delivered as follows:

Story, J. We are of opinion that the indictment upon these admitted facts cannot be maintained. The prisoner was unquestionably insane at the time of committing the offence. And the question made at the bar is, whether insanity whose remote cause is habitual drunkenness, is or is not an excuse in a court of law for a homicide committed by the party, while so insane, but not at the time intoxicated or under the influence of liquor. We are clearly of opinion that insanity is a competent excuse in such a case. In general, insanity is an excuse for the commission of any crime, because the party has not the possession of his reason, which includes responsibility. An exception is when the crime is committed by a party while in a fit of intoxication, the law not permitting a man to avail himself of the excuse of his own gross sin and misconduct to shelter himself from the legal consequences of such crime. But the crime must take place and be the immediate result of the fit of intoxication, and while it lasts, and not as in this case a remote consequence, superinduced by the antecedent exhauston of the party, arising from gross and habitual drunkenness. However criminal, in a moral point of view, such an indulgence is, and however justly a party may be responsible for his acts arising from it to Almighty God, human tribunals are generally restricted from punishing them, since they are not the acts of a reasonable being. Had the crime been committed while Drew was in a fit of intoxication he would have been liable to be convicted of murder. As he was not then intoxicated, but merely insane from an abstinance from liquor, he cannot be pronounced guilty of the offence. The law looks to the immediate and not to the remote cause, to the actual state of the party, and not to the cause which remotely produced it. Many species of insanity arise remotely from what, in a moral view, is a criminal neglect or fault of the party, as from religious melancholy, undue exposure, extravagant pride, ambition, &c. &c., yet such insanity has always been deemed a sufficient excuse for any crime done under its influence.

The jury without retiring from their seats, returned a verdict of *not guilty*. The case was conducted for the government by *George Black*, Esq. District Attorney; for the prisoner, by *Daniel Davis* and *Francis Bassett*, Esquires.

The exalted reputation of Judge Story, must give great authority to this opinion in our criminal courts generally.

We have also had the gratification to receive a letter from Professor Beck, the learned author of the System of Medical Jurisprudence, expressing his perfect accordance with our views of Birdsell's case, and of the moral character of *Mania a potu*. The following are his words:

I have read your papers on the case of Birdsell, with perfect conviction as to the correctness of your opinion on the subject of *Delirium Tremens*. And as far as I know, you are entitled to the further praise of originality in suggesting it. I confess myself also liable to the objection you have made, concerning the case of Mc. Donough, but at the time of writing, I had my mind so filled with the legal doctrine, as to the non-excusableness of actions committed while in a state of intoxication, that I too much overlooked his chronic disease and its effects.

It appears from the message of Governor Trimble to the General Assembly of Ohio, in December last, that Birdsell still remained in a state of mental alienation; a fact of which we have been assured by observations made by others as late as February. It is creditable to our chief magistrate, that he should have had the sagacity and firmness, to exercise, in favour of this convict, the prerogative of commutation of punishment; and thus preserve our judiciary from the odium of having illegally inflicted the punishment of death.

